# Identification and Pilot Evaluation of Salivary Peptides from *Anopheles albimanus* as Biomarkers for Bite Exposure and Malaria Infection in Colombia

**DOI:** 10.3390/ijms21030691

**Published:** 2020-01-21

**Authors:** Berlin Londono-Renteria, Papa M. Drame, Jehidys Montiel, Ana M. Vasquez, Alberto Tobón-Castaño, Marissa Taylor, Lucrecia Vizcaino, Audrey E. Lenhart

**Affiliations:** 1 Entomology Department, Vector Biology Laboratory, Kansas State University, 1603 Old Claflin Pl, 123 Waters Hall, Manhattan, KS 66506, USA; jehidys.montiel@udea.edu.co; 2 Department of Global Health, Duke University, 310 Trent Drive, Durham, NC 27710, USA; papa.drame@duke.edu; 3 Calle 70 No. 52–21, Malaria Group, Universidad de Antioquia, Medellin, Antioquia 05001, Colombia; amvc.ana@gmail.com (A.M.V.); alberto.tobon1@udea.edu.co (A.T.-C.); 4 Division of Parasitic Diseases and Malaria, Entomology Branch, Centers for Disease Control and Prevention (CDC), Atlanta, GA 30329, USA; marissaltaylor1@gmail.com (M.T.); vtb6@cdc.gov (L.V.); ajl8@cdc.gov (A.E.L.)

**Keywords:** *Anopheles albimanus*, salivary gland proteins, antibodies

## Abstract

Insect saliva induces significant antibody responses associated with the intensity of exposure to bites and the risk of disease in humans. Several salivary biomarkers have been characterized to determine exposure intensity to Old World *Anopheles* mosquito species. However, new tools are needed to quantify the intensity of human exposure to *Anopheles* bites and understand the risk of malaria in low-transmission areas in the Americas. To address this need, we conducted proteomic and bioinformatic analyses of immunogenic candidate proteins present in the saliva of uninfected *Anopheles albimanus* from two separate colonies—one originating from Central America (STECLA strain) and one originating from South America (Cartagena strain). A ~65 kDa band was identified by IgG antibodies in serum samples from healthy volunteers living in a malaria endemic area in Colombia, and a total of five peptides were designed from the sequences of two immunogenic candidate proteins that were shared by both strains. ELISA-based testing of human IgG antibody levels against the peptides revealed that the transferrin-derived peptides, TRANS-P1, TRANS-P2 and a salivary peroxidase peptide (PEROX-P3) were able to distinguish between malaria-infected and uninfected groups. Interestingly, IgG antibody levels against PEROX-P3 were significantly lower in people that have never experienced malaria, suggesting that it may be a good marker for mosquito bite exposure in naïve populations such as travelers and deployed military personnel. In addition, the strength of the differences in the IgG levels against the peptides varied according to location, suggesting that the peptides may able to detect differences in intensities of bite exposure according to the mosquito population density. Thus, the *An. albimanus* salivary peptides TRANS-P1, TRANS-P2, and PEROX-P3 are promising biomarkers that could be exploited in a quantitative immunoassay for determination of human-vector contact and calculation of disease risk.

## 1. Introduction

In spite of a significant decrease in malaria cases over the past decade, malaria is still an important public health concern in the Americas. *Plasmodium falciparum* accounts for ~25% of infections, with the majority of cases reported due to infections with *P. vivax* (~74.1%) [1] (WHO, 2018). Although uncommon, mixed infections are also present. In Colombia, malaria exhibits unstable epidemic/endemic patterns characterized by differing intensities and segregation between regions [2,3]. This diversity in malaria transmission is favored by the variety of geographic regions with differing climates and abundance variety of anopheline vectors [4]. *An. albimanus*, *An. Darlingi*, and *An. nuñeztovari* are the vectors considered responsible for the majority of malaria transmission in Colombia [5,6,7]. As in the rest of the continent, most malaria cases in Colombia are caused by *P. vivax* (70%). However, along the Pacific coast, *P. falciparum* is predominant and is associated with the largely Afro-Colombian communities with many Duffy-negative individuals [2].

Exposure to malaria parasites has traditionally been estimated using entomological and parasitological methods. However, these methods are labor-intensive and difficult to sustain in areas with low/unstable malaria transmission or in pre-elimination contexts [8,9] where the number of asymptomatic and submicroscopic carriers can be high [10] and hard to detect using classic microscopy-based parasitological methods. In such areas, it is tedious and labor-intensive to catch mosquitoes and rare to find them infected with *Plasmodium* sporozoites; consequently, entomological inoculation rates can be very low and do not often accurately reflect the transmission intensity. Thus, the development of new tools to reliably assess human exposure to bites from malaria vectors will improve our ability to monitor changes in malaria transmission risk over time at both population and individual levels.

The study of human–*Anopheles* immunological interactions has provided a promising basis for the development of tools that can quantify human exposure to vector bites. *Plasmodium* spp. are transmitted to humans in the saliva of infected female *Anopheles* spp. during the blood meal intake [11]. After being bitten by a mosquito (regardless of malaria infectiousness), humans produce immunoglobulin G (IgG), M (IgM), and/or E (IgE) specific to injected mosquito salivary proteins [12,13]. Such humoral responses may provide a sensitive marker of human exposure to vector bites and allow for estimating pathogen transmission risk associated with mosquito-borne diseases in various settings [14,15]. Indeed, all previous studies have described a correlation between the levels of anti-mosquito saliva antibodies in human blood and either levels of exposure to mosquito bites or the intensity/prevalence of mosquito-borne pathogens. In addition, such humoral responses have also shown an association with malaria severity [16]. However, the use of whole vector saliva is limited by potential cross-reactivities with salivary epitopes of other hematophagous arthropods, a lack of reproducibility between saliva batches and an inadequate production capacity for large-scale studies.

Recent progress in sialotranscriptomic research [17] has allowed for the identification of more specific antigens, enhancing the accuracy of mosquito “salivary” biomarkers. Notably, antibody responses to gSG6 or cE5, two *Anopheles* genus-specific proteins, represent reliable indicators of human exposure to *Anopheles* bites and the subsequent risk of malaria transmission [18,19]. Additionally, the gSG6-P1 peptide, designed from the *An. gambiae* gSG6 protein, has been described as suitable mosquito bite exposure biomarker due to its ability to ensure a high degree of specificity and reproducibility without losing sensitivity [20]. However, these protein/peptides biomarkers only share significant sequence homology with the Old World anophelines (Subgenus *Cellia*) and with mosquitoes from the subgenus Anopheles, which are globally distributed. However, gSG6 is absent in the subgenus *Nyssorhynchus* that harbors the major malaria vectors is Latin-American, *An. darlingi* and *An. albimanus* [17]. Therefore, developing a salivary peptide biomarker strategy for the New World anophelines requires exploratory research to identify appropriate markers. These biomarkers would be especially useful for the evaluation of malaria exposure risk in low-endemic settings, particularly when identifying how to best protect populations from malaria exposure as countries in the Americas intensify their efforts to achieve malaria elimination.

Previous studies suggest that vertebrate hosts exert an evolutionary pressure on arthropods salivary proteins [21]. Since insects maintained in colony are often fed with a restricted number of blood sources, and sometimes the blood has been processed (inactivated or defibrinated), we wanted to evaluate whether colonization affects salivary composition of mosquito from a long-maintained colony strain (STECLA) versus a recently colonized strain (Cartagena) [22] with the purpose of identifying variations in salivary content and identifying proteins that could be more closely related to exposure to the main vector bites in the field. Thus, salivary gland homogenate from these mosquitoes was used to identify the template of immunogenic candidates for the design of peptides with the potential to function as biomarkers of malaria infection risk in Central and South America. This study represents the first evaluation of *An. albimanus* salivary peptides as potential biomarkers for vector–human contact and risk of malaria in South America.

## 2. Results

### 2.1. Immunogenic Candidates Identified in the Sialome of An. Albimanus

**Figure 1:**
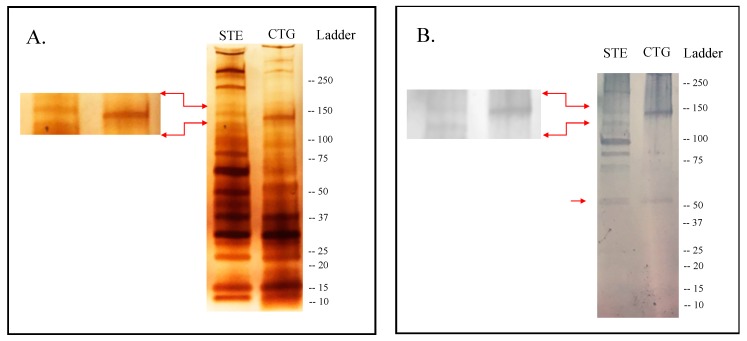
Band profile of salivary proteins contained in whole salivary gland extract (SGE) of *An. albimanus* strains STECLA (STE) and Cartagena (CTG). (**A**) Silver stain of proteins highlighting the ~140 kDa band absent in STE. (**B**) Immunoblotting with serum samples from healthy volunteers living in a malaria endemic area, highlighting the high reactivity against the ~140 kDa band in the CTG strain as well as the reactivity to a ~65 kDa band in both strains.

Silver-staining SDS-PAGE analysis of the SGE from adult females of the two *An. albimanus* strains showed slightly different protein profiles. Approximately 13–14 protein bands were identified in the SGE from STE, while ~11 were detected in CTG (Figure 1). At least three protein bands of 210 kDa and 140 kDa were observed in STE but not in CTG (Figure 1A). The immunoblotting analysis showed that human serum from healthy individuals recognized at least seven bands in the SGE from STE and two bands from CTG. A band of approximately 65 kDa was observed in both *An. albimanus* strains, while a band of ~140 kDa was observed only in CTG (Figure 1B).

Gel pieces were excised from areas corresponding to the 65 kDa immunogenic band in the western blot, identified in two independent experiments, and each one was sent for mass spectrometry analysis. Protein sequencing results produced a total of nine proteins with molecular weights between 59 kDa and 65 kDa. Five of these proteins were shared by the two strains, one was specifically found in the STE strain while three were uniquely identified in the CTG (Table 1). Although it is possible that not all these proteins are immunogenic, seven out of nine identified proteins have a signal peptide sequence, suggesting that they are likely to be secreted. We are considering them as potential candidates for the immunogenic band found in the western blot.

Among the proteins shared by both strains we found a ferritin (A0A182FAJ2, VectorBase ID AALB003521) [23], a member of the 5′ nucleotidase, (A0A182FTN8, VectorBase ID AALB009922), a member of the salivary apyrase-5′nucleotidase family previously described in other Culicidae members [24,25]. A 1-pyrroline-5-carboxylate dehydrogenase, (A0A182FP42, VectorBase ID AALB008305) involved in the synthesizes L-glutamate. A heme-binding protein with peroxidase activity (A0A1Y9G8H0, VectorBase ID AALB016040) that shares 99% homology with the Q9XYP9 (VectorBase ID AALB016040) a salivary peroxidase previously characterized in *An. albimanus* mosquitoes [26] and identified in our current work only on the STE SGE sample, suggesting that these two proteins are very likely the same protein. Finally, we found the A0A1Y9G8K4 (VectorBase ID AALB016037), another uncharacterized heme-binding protein with predicted salivary protein with peroxidase activity and associated with responses against oxidative stress.

In the case of the recently colonized CTG strain, we found A0A182FH19 (VectorBase ID AALB005812), an uncharacterized protein predicted to belong to the phosphohexose mutase family. We also found A0A1Y9G9T7 (VectorBase ID AALB016038) and A0A1Y9G9L7 (VectorBase ID AALB016039), two more heme-peroxidases that, along with the Q9XYP9 and A0A1Y9G8K4, form a cluster on chromosome 3R [17], suggesting that heme peroxidases are an important group of immunogenic proteins in *An. albimanus* salivary proteins with high potential as markers of exposure to bites.

### 2.2. Selection of Candidate Biomarkers

Since *An. albimanus* is spread through Central and South America, we designed peptides that were common to both strains to maximize their geographical relevance. Thus, after filtering the data for secreted proteins with little or no sequence homology with human and other Culicids we selected two proteins shared by STE and CTG and designed two peptides for each protein: A0A182FAJ2, a transferrin (TRANS-P1 and TRANS-P2); A0A1Y9G8H0, an uncharacterized protein with potential activity as a peroxidase (PEROX-P1 and PEROX-P2); and Q9XYP9, a salivary peroxidase that was only identified in STE (PEROX-P3) but has >99% of similarity with the A0A1Y9G8H0 and very likely represent the same protein. Thus, we designed two peptides for the transferrin sequence and three peptides mapping the salivary peroxidases to limit the in silico specificity to New World anophelines. Details of these proteins as well as the peptide sequences are presented in Table 2.

In addition, the uncharacterized proteins A0A1Y9G8K4 (VectorBase ID AALB016037), A0A1Y9G9T7 (VectorBase ID) AALB016038) and A0A1Y9G9L7 (VectorBase ID AALB016039), form a cluster on chromosome 3R along with the Q9XYP9, with which they show between 51% and 73% of identity [17]. They are all members of the peroxidase family, suggesting that members of this family in *An. albimanus* are highly recognized by antibodies in serum of people exposed to bites from these mosquito species.

### 2.3. Human Antibody Responses Specific to An. albimanus Salivary Peptides

**Figure 2:**
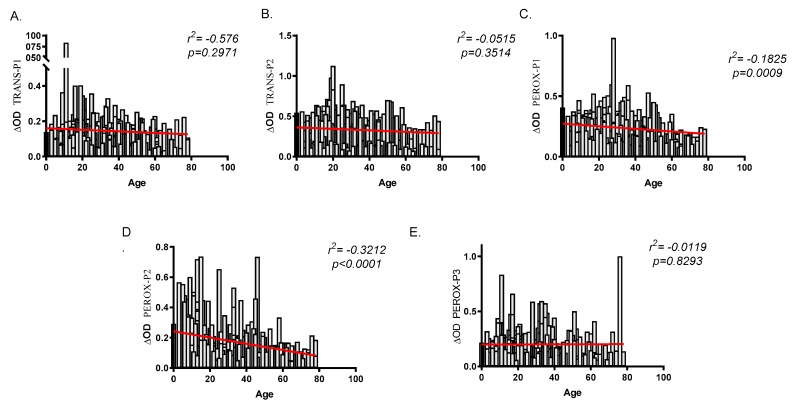
Correlation analysis between age and IgG antibody levels against each one of the salivary peptides (**A**–**E**). Spearman rank correlation was used to test significance with a *p* < 0.05.

To validate the peptides as biomarkers for human exposure to *An. albimanus* bites and risk of malaria infection, we evaluated the total IgG antibodies in serum samples originating from two malaria endemic areas with different densities of *An. albimanus*—El Bagre (lower *An. albimanus* abundance) and Turbo (higher *An. albimanus* abundance). A total of 337 samples, 185 from El Bagre and 152 from Turbo were analyzed with an age range between three and 78 years old in Turbo (mean 18.8) and between three and 74 years old (mean 28.65) in El Bagre. Among the study participant 3.29% were children under five years old in Turbo (*n* = 5) and 1.62% in El Bagre (*n* = 3). Malaria diagnosis by microscopy identified a total of 68 malaria positive and 117 malaria negative samples from El Bagre and 45 malaria positive and 107 malaria negative samples from Turbo. Analysis of the antibody levels against all of the peptides showed a negative correlation between age and IgG antibodies, although this correlation was only significant when comparing age and the IgG antibody levels against PEROX-P1 (*r*^2^ *=* −0.1825, *p* = 0.0009) and PEROX-P2 (*r*^2^ = −0.3212, *p* = 0.0000) (Figure 2). Moreover, when comparing antibody levels between males and females, we only observed significantly higher IgG antibody levels against TRANS-P2 (*p* = 0.0118) in females (data no shown).

**Figure 3:**
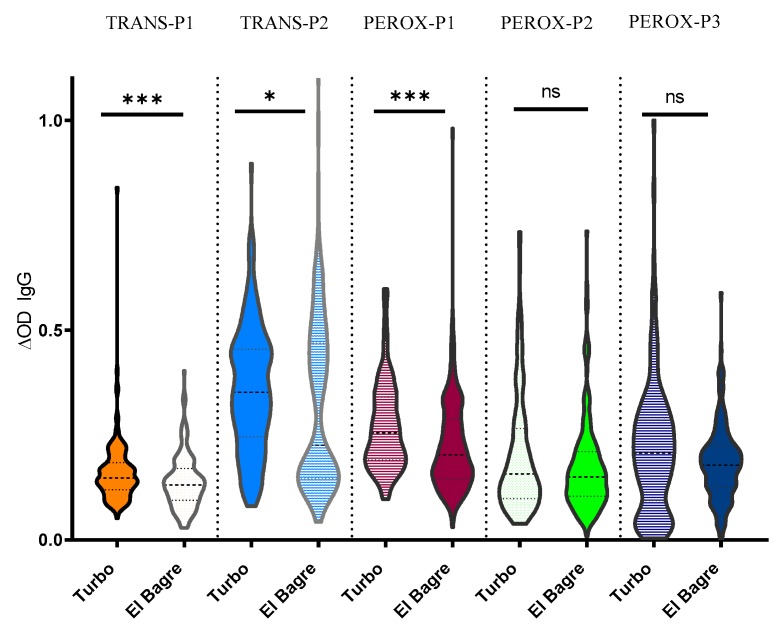
Schematic representation of the IgG antibody levels against each peptide by location Pairwise significance was tested with Mann-Whitney test with significance represented by stars (*p <* 0.05 (* = 0.01 to 0.05, ** = 0.001 to 0.01, and *** = 0.0001 to 0.001). ns denotes “not significant.”

Since previous studies suggested that Turbo and El Bagre presented differences in the abundance of *An. albimanus* [7,27], we evaluated whether there were any differences in the antibody levels against the peptides in these areas that could be associated with mosquito abundance and vector–human contact. We observed that IgG antibody levels were higher in Turbo; however, pairwise comparisons show that the difference was not significant for IgG antibody levels against PEROX-P2 and PEROX-P3 (Figure 3).

**Figure 4:**
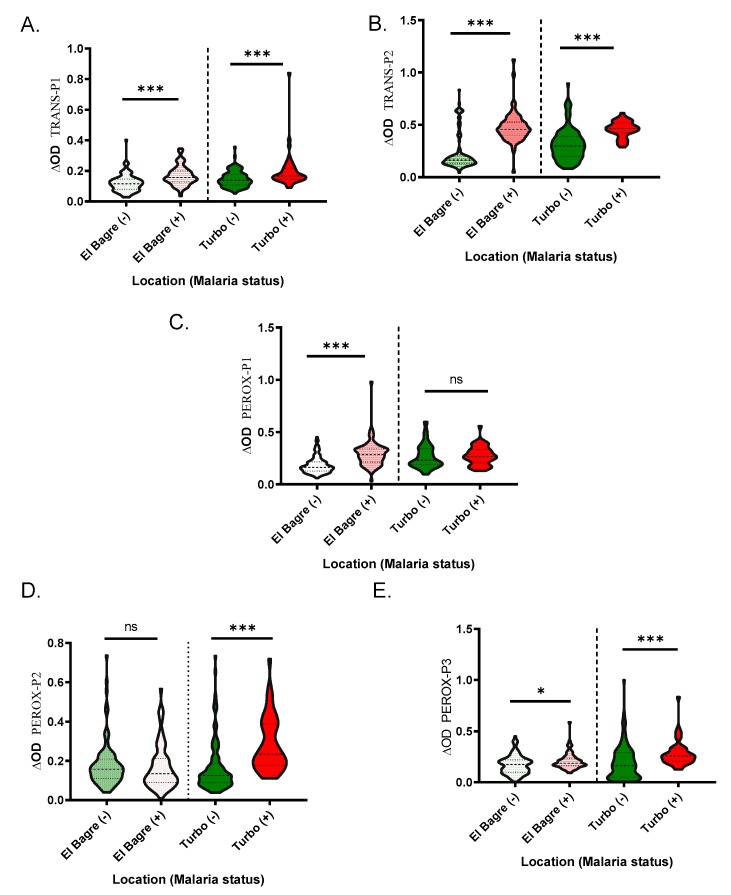
Schematic representation of the IgG antibody levels against the *An. albimanus* peptides, TRANS-P1 (**A**), TRANS-P2 (**B**), PEROX-P1 (**C**), PEROX-P2 (**D**), and PEROX-P3 (**E**). Pairwise significance was tested with Mann-Whitney test with significance represented by stars (*p <* 0.05 (* = 0.01 to 0.05, ** = 0.001 to 0.01, and *** = 0.0001 to 0.001). ns denotes “not significant.”

When comparing IgG antibody levels and malaria infection status, we found that samples with active malaria infection had significantly higher antibody levels than the controls for all the peptides, with the exception of the IgG levels against PEROX-P1 in Turbo (*p* = 0.7614) and PEROX-P2 in El Bagre (*p* = 0.5343) (Figure 4).

**Figure 5:**
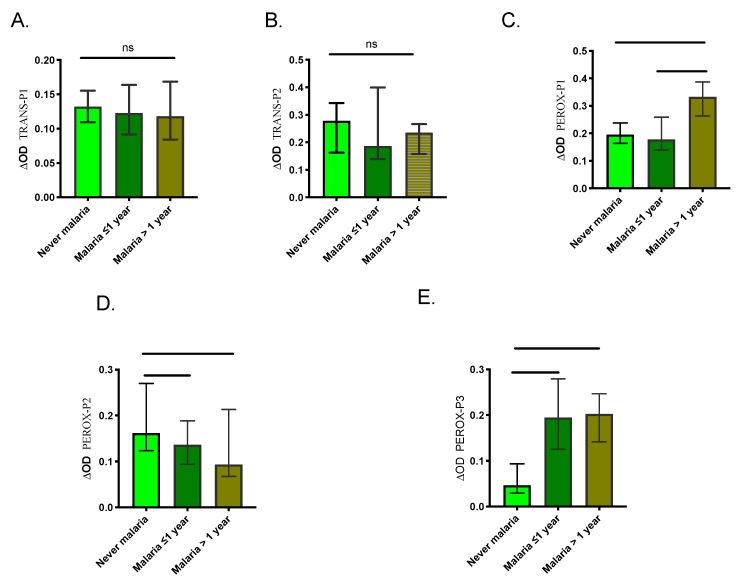
Graphic representation of the IgG antibody levels against each peptide (**A**–**E**) in samples originating from individuals with a negative malaria diagnosis from each control group. Significance was tested with the Kruskal-Wallis test and Dunn’s multiple comparison test with significance represented by stars (*p* < 0.05 (*=0.0, **=0.00 and ***=0.000). ns denotes “not significant.”

We also compared the level of antibodies between the three control groups to assess the ability of the peptides to discriminate previous exposure to malaria. Our results showed no significant differences in IgG levels among the control groups for TRANS-P1 and TRANS-P2 (Figure 5). However, the malaria >1-year group presented significantly higher IgG antibodies against PEROX-P1 while never malaria group showed significantly higher antibodies against PEROX-P2. Also, never malaria group presented significantly lower antibodies against the PEROX-P3 than the other groups.

## 3. Discussion

Due to the current efforts to control and eliminate malaria, the trend in cases has significantly decreased in most parts of the world. However, in Latin America, the WHO reported an increase in malaria infections by *P. vivax* [1] (WHO, 2018). In Colombia, the malaria situation is also complex. In the past five years, an overall decrease in areas of high transmission has been observed while an increase of malaria cases has been observed in areas historically lower for malaria transmission or where the parasite has been introduced due to factors like migration and changes in environment [28]. Thus, tools to evaluate vector-human contact and the protective efficacy of vector control interventions are now more important than ever in the fight for malaria elimination.

Current methods to evaluate the risk of malaria transmission and the impact of malaria intervention programs are less sensitive and less effective in low-endemic, epidemic or unstable transmission settings [29]. A highly sensitive point-of-care field test is needed to rapidly detect low-density parasitemia and identify all infected individuals, in order to enable immediate treatment and more accurate surveillance. Addressing this need, we have identified two immunogenic candidate proteins present in the secretome of two strains of *An. albimanus* by combining 1D electrophoresis, western blot and mass spectrometry approaches. Saliva and salivary gland composition depend of a wide range of factors including infectious status, age, environment and blood feeding status [21]. We used blood fed, 8–10 days old female mosquitoes in our experiment to keep comparability of the two SGE comparable.

Interestingly, more protein bands with higher immunogenicity were identified when using SGE from the *An. albimanus* STE strain compared to the CTG strain. The pattern was very similar to the one previously reported by our group, where proteins with molecular weights higher than 75 kDa were identified by antibodies in serum from exposed volunteers living in Haiti [13]. Several factors may be associated with the observed differences. In our most recent studies, we found discreet differences in the salivary gland protein composition and immunogenicity of the lone star tick, *Amblyomma americanum* when comparing ticks maintained in colony from those that were recently captured from the field [30]. Previous studies also suggest that host-vector interactions may exert an immunological pressure that may shape salivary content in certain arthropod groups [31,32]. Regardless of the differences, the existence of common immunogenic candidate protein shows that common salivary protein epitopes are shared between these two strains, indicating the possibility of developing a salivary biomarker of *An. albimanus* bites that is applicable across its geographical range. Based on the sequences of these proteins, we developed an ELISA based assay for five immunogenic peptides from two salivary proteins found in SGE of *An. albimanus*, that very likely are recognized by other Anopheles from the New World. Thus, although further and exhaustive studies are needed, we propose these peptides as potential biomarkers of malaria transmission intensity and risk in Latin America and the Caribbean.

The majority of the *An. albimanus* salivary proteins selected as candidate biomarkers have significant sequence homology with another New World anopheline, such as *An. darlingi* and *An. triannulatus*. As such, salivary proteins triggering the human host immune response seem to be well-conserved among species of the New World *Nyssorhynchus* subgenus, as described for some salivary protein families [17]. This would, in theory, allow for the identification of a unique biomarker capable of assessing the level of human exposure to all potential malaria vector bites and the associated risk of malaria infection. Such a biomarker could be particularly useful in regions of the Americas where malaria transmission occurs by multiple vector species that exist in sympatry [33]. A similar approach has led to the identification and validation of the gSG6 and cE5 salivary proteins of *An. gambiae* as biomarkers of exposure to *Anopheles* bites and risk of malaria transmission in different African settings [34]. To date, there are not any reports of immunogenic peptides developed to evaluate exposure to bites from New World *Anopheles*. In our study, all the tested peptides from proteins identified from a ~65 kDa band showed to be immunogenic in western blot. Then, the most probable immunogenic candidates from that band were selected to design peptides. Importantly, the 59 kDa-transferrin peptides (TRANS-P1 and P2) showed capacity to distinguish infected from uninfected individuals and hold great promise as potential biomarkers of malaria infection risk.

In insects, transferrin has been associated with anti-microbicide effect [35] and with antioxidant properties [36]. Interestingly, transferrin synthesis and secretion have been shown to increase on exposure of Aedes mosquito cells to pathogens [35], suggesting that mosquito transferrin participates as an acute-phase protein that is up-regulated during the mosquito immune response. Although, no upregulation of transferrin has been observed in Anopheles mosquitoes, we hypothesize that higher levels of transferrin (and other structural proteins) from the mosquito salivary gland secretory cells may be deposited in the secretory canal during the travel of the sporozoite from the mosquito to the human. Previous studies have shown that sporozoites have to enter and leave several salivary gland cells before they maturate and reach the secretory canal [37], and that discreate level of disruption and cell death in salivary gland may be caused by the presence of sporozoites [38]. This could explain why IgG antibody levels against TRANS-P1 and P2 peptides were higher in the samples originating from infected individuals. Is it possible that several normally “non-secreted” protein reach saliva of infected mosquitoes during this process? More studies are needed to evaluate the content on *An. albimanus* pure saliva of infected mosquitoes from the field, how transferrin is associated with malaria infection, and to evaluate gene expression patterns of transferrin during malaria infection in New World Anopheles. In addition, people with active malaria presented higher antibody levels against these transferrin peptides, and the absence of significant differences in IgG levels against the transferrin peptides among the samples originating from uninfected individuals who never had malaria or had malaria in the past (before the study sampling) strengthens that hypothesis.

In this study, we designed three peptides from salivary peroxidases. A ~66 kDa salivary peroxidase has been previously reported in *An. albimanus* salivary homogenates associated with relaxation of smooth muscle [26]. Salivary peroxidases have also been associated with probing and blood feeding [39]. *An. albimanus* has been described as the main malaria vector in Turbo while *An. darlingi* was the most abundant species transmitting malaria in El Bagre [27,40], which may explain the tendency for higher IgG antibody levels against *An. albimanus* peptides in Turbo. However, with the exception of PEROX-P2 and PEROX-P3, the differences in antibody levels between malaria infected and non-infected groups varied according to location. Our results suggest that the level of IgG antibodies against each peptide is associated with the individual immunogenicity of each peptide and regardless of whether the bites were infective. We found a significant negative correlation between age and the peptides PEROX-P1 and PEROX-P2. A decrease in antibodies with age may be associated with the induction of tolerance against chronic exposure to these antigens [13,15]. Aggressiveness and biting behaviors can differ between species, resulting in a difference in the level of human-vector contact. The application of immunoassays based on these peptides could help discern epidemiological variations in the risk of malaria infection and help to better guide strategies for malaria control and elimination.

It is important to specify that, in the field majority >90% of the bites are from uninfected mosquitoes and that antibodies against salivary proteins are short lived. Thus, an interesting result from our study is the lower level of IgG antibodies against the PEROX-P3 in samples originating from individuals that had never experienced malaria even when they are residents or malaria endemic areas, suggesting that changes in antibody levels against this peptide may be an important marker of recent exposure to New World *Anopheles* bites in otherwise naïve populations (i.e., deployed military personnel and travelers). However, further testing with a sufficiently powered human population is necessary to test this hypothesis.

This study has several limitations. Ideally, serum samples originating from individuals that are infected with other mosquito-borne diseases like dengue as well as samples from people living in non-endemic areas who have never been exposed to mosquito bites should be tested. It is important to make emphasis that the selection of the candidates was based on western-blot and the in silico detection of signal peptides rather than the fact that the proteins appear in different strains. To further validate the IgG antibodies against PEROX-P3, TRANS-P1, and TRANS-P2 as candidate biomarkers, the peptides should be evaluated together with entomological inoculation rates. Also, there is a potential for comigration of proteins leading to false identification of proteins specially when the 1D electrophoresis was used for protein separation. However, we believe that further confirmation of the immunogenicity of he selected candidates is demonstrated by the ELISA results. Finally, it will be important to evaluate these peptides before and after vector control activities to evaluate their efficacy in detecting protection from *Anopheles* bites as a result of vector control interventions. Ultimately, we hope to validate biomarkers that can be readily applied in public health surveillance efforts to measure the intensity of exposure to infective bites and to evaluate the protective efficacy of vector control interventions.

## 4. Materials and Methods

### 4.1. Study Site and Serum Samples for Validation of Biomarkers

Serum samples from 337 participants were randomly selected for use in the biomarker validation (Table 3). The samples used to validate the biomarkers were collected as part of a malaria study in Colombia. Human serum samples were collected by active and passive case detection in a transversal phase study, conducted from November 2016 to October 2017 in two malaria endemic areas of Colombia with different malaria incidence, Turbo (8.0952° N, 76.7285° W) and El Bagre (7.6057° N, 74.8063° W) located in Antioquia Department, northwest Colombia. Specifically, Turbo has a low Annual Parasite Incidence = (confirmed cases during 1 year/population under surveillance) × 1000; 1.22 and 0.77 in 2016 and 2017 respectively, while the malaria incidence in El Bagre was higher with an API of 24, 78 and 21.3 during the same period of time (2016 and 2017, respectively) [41]. Malaria transmission in Colombia occurs year-round, with peaks typically occurring between February and June [4]. The methods and protocols were reviewed and approved by the Ethics Committee at Medicine School, Universidad de Antioquia in Medellín, Colombia (Record 011 dated 28 July 2016) and by the Kansas State University Institutional Review Board (IRB 1206).

Regarding the *Anopheles* distribution in the study places, Gutiérrez et al. (2009) [7] reported that in Turbo, *An. albimanus* was the most abundant species (96.6%), although other species were present at ≤1.1% (*An. nuneztovari*, *An. pseudopunctipennis*, *An. Punctimacula*, and *An. neomaculipalpus*). In El Bagre, the two most common mosquito species are *An. darlingi* (71.9%) and *An. braziliensis* (20.3%), with minor malaria vectors (<2.5%) including *An. nuneztovari*, *An. triannulatus*, *An. punctimacula*, *An. neomaculipalpus*, and *An. albitarsis* [7]. The human biting rate (HBR) for *An. albimanus* in the region varies between 0.153–0.457, while the HBR of *An. darlingi* is lower, between 0.040 and 0.163) [7].

Malaria diagnosis was done by microscopy and confirmed by nested PCR (nPCR). DNA was extracted from half-blood spot filter (approximately 30 µL of blood) using QIAamp DNA Mini Kit (Qiagen, Germany), according to manufacturer’s instructions. nPCR was performed as a two-step procedure, using 2 µL of DNA template and following the protocol described by Singh et al. [42]. Amplification products were resolved in a 1.5% agarose gel stained with GelRed™ (Biotium, Fremont, CA, USA) and visualized under UV light. Samples were classified as malaria positive or malaria negative. Malaria negative samples were further classified as “never malaria” (originating from individuals who had never had malaria), “Malaria ≤ 1 year” (originating from individuals who had malaria in the last year before the sampling date), and “malaria >1 year” (originating from individuals whose last malaria episode was more than a year before the sampling date).

### 4.2. Mosquito Rearing and Salivary Gland Extraction

Two *An. albimanus* insectary strains were used in this study, STECLA (STE) and Cartagena (CTG), which were maintained in the insectary at the US Centers for Disease Control and Prevention (CDC, Atlanta, GA, USA). Salivary glands from female mosquitoes were extracted by dissection and pooled into 1× PBS [13]. Female mosquitos were 8–10 days old, blood feed day 3 or 4. A pool of 100 salivary gland pairs from each strain was frozen and thawed three times to prepare the Salivary Gland Extract (SGE). SGE protein concentration was determined using a NanoDrop™ (Thermo Scientific, Wilmington, DE, USA) and 50 µL aliquots were stored at −80 °C until use.

### 4.3. SDS and Western Blot

For SGE from both CTG and STE, 5 µg of sample was diluted in an equal volume of 2× laemmli sample buffer (Bio-Rad, Hercules, CA, USA), and the mix was boiled at 95 °C for 5 min in a thermomixer and then centrifuged at 16,000× *g* for 1 min. The supernatant of each diluted sample was loaded into the wells of a mini 4–12% gradient acrylamide gel, along with pre-stained protein molecular weight markers (Bio-Rad, Hercules, CA, USA). Each sample was run in duplicate and protein separation was done in a ready-gel Tris-HCl buffer (Bio-Rad) at 120 volts for about 1 h. Gels were then washed with PBS, and the proteins were visualized using the Silver Stain kit (Thermo Scientific, Waltham, MA, USA) according to the manufacturer’s instructions.

The separated proteins were transferred onto a polyvinylidene difluoride (PVDF) membrane using the Trans-Blot Turbo semi-dry blotting transfer system (Bio-Rad) by selecting the rapid transfer option (25 V, 2.5 A and 15 min). After washing three times with PBS containing 0.05% Tween 20 (washing buffer), the membrane was blocked with 2% skim milk diluted in washing buffer (blocking buffer) for one hour at room temperature. Then, the membrane was washed three times and immunogenic bands were detected after an overnight incubation at 4 °C of the membrane with a pool of 10 human sera samples from individuals with a history of malaria but uninfected during the sample collection (five individuals come from Turbo and five from El Bagre) from individuals living in a malaria endemic area (1:100 dilution in blocking buffer). Detection of specific IgG antibodies was performed using a horseradish peroxidase (HRP) conjugated goat anti-human IgG secondary antibody (Abcam, Cambridge, MA, USA) added at a dilution of 1:1000 in blocking buffer and incubated for one hour at room temperature. After incubation with the secondary antibody, the membrane was washed three times and incubated with TMB (Abcam) until a desired level of staining was achieved, and the reaction was stopped by washing the membrane several times with ultrapure water.

### 4.4. Peptide Design and Selection

Gel immunogenic protein bands were cut and sent for identification to Bioproximity LLC (Manassas, VA, USA). Global proteomic profiling was acquired using ultraperformance liquid chromatography and tandem mass spectrometry. Selected taxa for the identity of the peptides was Anopheles genus. Briefly, gel band samples were prepared using suspension-trapping (S-trap, Protifi) (Huntington, NY, USA) for digestion and the digested peptides were collected by centrifugation. Peptides were eluted with 80% acetonitrile, 5% ammonium hydroxide, and lyophilized in a SpeedVac (ThermoFisher Scientific, Waltham, MA, USA) in order to remove volatile components. UHPLC-MS/MS was used to analyze the digestion mixtures. LC was performed on an Easy-nLC 1000 UHPLC system (ThermoFisher Scientific) interfaced to a quadrupole-Orbitrap mass spectrometer (Q-Exactive HF-X, Thermo Fisher) via nano-electrospray ionization using a source with an integrated column heater, Thermo Easy Spray source (ThermoFisher Scientific) [43,44,45]. Identified proteins were analyzed for the presence of signal peptide (signal P) sequence using SignalP 5.0 server and for sequence homologies (at least 50% identity with E-value 1 × 10^−5^) to human and other major culicid disease vector species using online BLAST program. A protein sequence and structure analysis were then performed for the top Anopheles-specific proteins using the Protean 3D package of the DNASTAR software (DNASTAR Inc., Madison, WI, USA). Also, the DNASTAR software was used for the analysis of B-cell epitopes, antigenic regions, flexible regions (turns), hydrophobic regions, stability and/or charge density by importing from UniProt or NCBI the FASTA files of sequences of interest. Highly antigenic, stable, flexible, charged and less than 50% hydrophobic regions that were specific to *Anopheles mosquitoes* (>50% homology) were selected using the software for 18–22 amino acid peptides. Peptide sequences were then sent for synthesis (Genscript, Piscataway, NJ, USA). All synthetic peptides were received in lyophilized form, dissolved in ultrapure water and frozen at −20 °C until use as antigens in ELISA assays.

### 4.5. Human IgG Detection by Indirect ELISA

Specific IgG responses in the human serum samples against salivary extract and peptides were measured by ELISA. Working conditions using the total SGE were based on previous studies. In the case of the ELISAs involving the testing of antibodies against the peptides, an optimization of working conditions was performed testing increasing serum dilutions (1/100 and 1/200). The optimal peptide concentration was evaluated measuring IgG antibody titles against 1, 2, 4, and 8 µg/mL. Based on the results from the titration, 96-well ELISA plates Nunc-Maxisorp plates (Nalgene Nunc International, Rochester, NY, USA) were coated with 50 µL/well of single synthetic peptide (2 μg/mL) in PBS and incubated overnight at 4 °C. Plates were blocked with 200 µL of 5% skim milk solution in PBS-Tween 20 (0.05%) for one hour at 37 °C. After washing the plates three times with PBS-Tween-0.1%, 50 µL diluted serum samples (1/100 in 5% milk in PBS-Tween-0.05%) was added to each plate well in duplicate and plates were again incubated at 37 °C for two hours. Plates were again washed three times before 50 µL of goat monoclonal anti-human IgG conjugated with horseradish peroxidase (AbCam, Cambridge, MA, USA) were added at 1/1000 dilution and incubated for one hour at 37 °C. After three final washes, colorimetric development was carried out using tetra-methyl-benzidine (AbCam) as substrate. The reaction was stopped with 0.25 N sulfuric acid and the optical density (OD) was measured at 450 nm. In parallel, each assessed microplate contained in duplicate: positive control (pool of ten human sera samples from individuals residing in both study areas infected with Plasmodium). Two wells coated with the antigen but without serum was taken as negative control. The blank represented wells with no antigen nor serum.

### 4.6. Statistical Analyses

Results were expressed as the ΔOD value:

ΔOD = ODx − ODb(1)

where ODx represents the mean of individual OD in both antigen wells and ODb the mean of the blank wells.

For each tested peptide, plate positive controls were averaged and divided by individual plate positive control ΔOD values to obtain a normalization factor for each plate as previously described [46]. Each plate normalization factor was multiplied by plate sample ΔOD to obtain normalized ΔOD values that were used in statistical analyses. Differences in observed values among more than two independent groups were assessed using the Kruskal-Wallis test. Pairwise comparisons between independent groups were tested using the Mann-Whitney test, and the association between independent groups was estimated by the non-parametric Spearman correlation method. Data were analyzed and graphs were constructed with GraphPad Prism8 software (San Diego, CA, USA).

## 5. Conclusions

We applied a proteomic approach to discover five new putative biomarkers of risk of malaria infection in the saliva of *An. albimanus* that were immunogenic in humans. Antibody levels against PEROX-P3, TRANS-P1, and TRANS-P2 were significantly higher in serum samples from malaria-infected individuals compared to samples from uninfected individuals, while a peroxidase found only in the STE strain was significantly lower in samples from individuals that were naïve to malaria infections. Therefore, the use these peptides as biomarkers of both exposure to New World *Anopheles* bites and malaria transmission risk could serve as important tools in malaria surveillance and control programs.

## Figures and Tables

**Table 1:** List of proteins found by mass spectrometry in a ~65 kDa protein excised from both *An. albimanus* strains (CTG and STE).

Protein ID	Description	CTG	STE	Length	Molecular Weight (Da)
A0A182FAJ2	Transferrin	YES	YES	532	59.390
A0A182FH19	Uncharacterized protein	YES	NO	561	60.603
A0A182FTN8	Uncharacterized protein	YES	YES	568	63.192
A0A182FP42	Uncharacterized protein	YES	YES	573	63.502
Q9XYP9	Salivary peroxidase	NO	YES	591	65.445
A0A1Y9G8H0	Uncharacterized protein	YES	YES	592	65.508
A0A1Y9G8K4	Uncharacterized protein	YES	YES	593	66.000
A0A1Y9G9T7	Uncharacterized protein	YES	NO	589	66.231
A0A1Y9G9L7	Uncharacterized protein	YES	NO	589	66.339

**Table 2:** List and characteristics of the immunogenic peptides.

Protein ID	Description	Length	Mass	Peptide Name	Peptide Sequence	Amino-Acid Position
A0A182FAJ2	Transferrin	532	59.390	TRANS-P1	YSPNADIDGLMKKRYSNL	185–202
				TRANS_P2	SYLCEDGTTRPVSDQNVC	271–288
A0A1Y9G8H0/Q9XYP9	Salivary peroxidase	592	65.508	PEROX-P1	RTITDCDADPSSCSNSKKAE	162–181
				PEROX-P2	MKVETRDGSDWPPRNPNAST	214–233
		591	65.445	PEROX-P3	QRARDHGLPSYNSFREKCGL	434–453

**Table 3:** Demographic description of the individuals from which serum samples originated, categorized by malaria diagnosis, malaria positive (+) or malaria negative (−), and sex.

Status	Turbo		El Bagre		Total
	Females	Males	Females	Males	
Malaria +	15	29	23	45	112
Malaria −	63	44	67	50	224
Total	78	73	90	95	336 *

* There is one missing data for the sex variable.

## References

[1] World Health Organization World Malaria Report 2018. http://www.who.int/iris/handle/10665/275867.

[2] Rodriguez J.C., Uribe G.A., Araujo R.M., Narvaez P.C., Valencia S.H. (2011). Epidemiology and control of malaria in Colombia. Memórias Inst. Oswaldo Cruz.

[3] Feged-Rivadeneira A., Angel A., Gonzalez-Casabianca F., Rivera C. (2018). Malaria intensity in Colombia by regions and populations. PLoS ONE.

[4] Montoya-Lerma J., Solarte Y.A., Giraldo-Calderon G.I., Quinones M.L., Ruiz-Lopez F., Wilkerson R.C., Gonzalez R. (2011). Malaria vector species in Colombia: A review. Memórias Inst. Oswaldo Cruz.

[5] Naranjo-Diaz N., Rosero D.A., Rua-Uribe G., Luckhart S., Correa M.M. (2013). Abundance, behavior and entomological inoculation rates of anthropophilic anophelines from a primary Colombian malaria endemic area. Parasit Vectors.

[6] Gutierrez L.A., Naranjo N., Jaramillo L.M., Muskus C., Luckhart S., Conn J.E., Correa M.M. (2008). Natural infectivity of Anopheles species from the Pacific and Atlantic Regions of Colombia. Acta Trop..

[7] Gutierrez L.A., Gonzalez J.J., Gomez G.F., Castro M.I., Rosero D.A., Luckhart S., Conn J.E., Correa M.M. (2009). Species composition and natural infectivity of anthropophilic Anopheles (Diptera: Culicidae) in the states of Cordoba and Antioquia, Northwestern Colombia. Memórias Inst. Oswaldo Cruz.

[8] Beier J.C., Killeen G.F., Githure J.I. (1999). Short report: Entomologic inoculation rates and Plasmodium falciparum malaria prevalence in Africa. Am. J. Trop. Med. Hyg..

[9] Drakeley C.J., Corran P.H., Coleman P.G., Tongren J.E., McDonald S.L., Carneiro I., Malima R., Lusingu J., Manjurano A., Nkya W.M. (2005). Estimating medium- and long-term trends in malaria transmission by using serological markers of malaria exposure. Proc. Natl. Acad. Sci. USA.

[10] Bousema T., Okell L., Felger I., Drakeley C. (2014). Asymptomatic malaria infections: Detectability, transmissibility and public health relevance. Nat. Rev. Microbiol..

[11] Ribeiro J.M., Francischetti I.M. (2003). Role of arthropod saliva in blood feeding: Sialome and post-sialome perspectives. Ann. Rev. Entomol..

[12] Orlandi-Pradines E., Almeras L., Denis de Senneville L., Barbe S., Remoue F., Villard C., Cornelie S., Penhoat K., Pascual A., Bourgouin C. (2007). Antibody response against saliva antigens of Anopheles gambiae and Aedes aegypti in travellers in tropical Africa. Microbes Infect..

[13] Londono-Renteria B.L., Eisele T.P., Keating J., James M.A., Wesson D.M. (2010). Antibody response against Anopheles albimanus (Diptera: Culicidae) salivary protein as a measure of mosquito bite exposure in Haiti. J. Med. Entomol..

[14] Remoue F., Cisse B., Ba F., Sokhna C., Herve J.P., Boulanger D., Simondon F. (2006). Evaluation of the antibody response to Anopheles salivary antigens as a potential marker of risk of malaria. Trans. R. Soc. Trop. Med. Hyg..

[15] Cardenas J.C., Drame P.M., Luque-Burgos K.A., Berrio J.D., Entrena-Mutis E., Gonzalez M.U., Carvajal D.J., Gutierrez-Silva L.Y., Cardenas L.D., Colpitts T.M. (2019). IgG1 and IgG4 antibodies against Aedes aegypti salivary proteins and risk for dengue infections. PLoS ONE.

[16] Andrade B.B., Rocha B.C., Reis-Filho A., Camargo L.M., Tadei W.P., Moreira L.A., Barral A., Barral-Netto M. (2009). Anti-Anopheles darlingi saliva antibodies as marker of Plasmodium vivax infection and clinical immunity in the Brazilian Amazon. Malar. J..

[17] Arca B., Lombardo F., Struchiner C.J., Ribeiro J.M. (2017). Anopheline salivary protein genes and gene families: An evolutionary overview after the whole genome sequence of sixteen Anopheles species. BMC Genom..

[18] Rizzo C., Ronca R., Fiorentino G., Mangano V.D., Sirima S.B., Nebie I., Petrarca V., Modiano D., Arca B. (2011). Wide cross-reactivity between Anopheles gambiae and Anopheles funestus SG6 salivary proteins supports exploitation of gSG6 as a marker of human exposure to major malaria vectors in tropical Africa. Malar. J..

[19] Stone W., Bousema T., Jones S., Gesase S., Hashim R., Gosling R., Carneiro I., Chandramohan D., Theander T., Ronca R. (2012). IgG responses to Anopheles gambiae salivary antigen gSG6 detect variation in exposure to malaria vectors and disease risk. PLoS ONE.

[20] Poinsignon A., Cornelie S., Ba F., Boulanger D., Sow C., Rossignol M., Sokhna C., Cisse B., Simondon F., Remoue F. (2009). Human IgG response to a salivary peptide, gSG6-P1, as a new immuno-epidemiological tool for evaluating low-level exposure to Anopheles bites. Malar. J..

[21] Coutinho-Abreu I.V., Guimaraes-Costa A.B., Valenzuela J.G. (2015). Impact of Insect Salivary Proteins in Blood Feeding, Host Immunity, Disease, and in the Development of Biomarkers for Vector Exposure. Curr. Opin. Insect Sci..

[22] Olano V.A., Carrillo M.P., de la Vega P., Espinal C.A. (1985). Vector competence of Cartagena strain of Anopheles albimanus for Plasmodium falciparum and P. vivax. Trans. R. Soc. Trop. Med. Hyg..

[23] Papa F., Windbichler N., Waterhouse R.M., Cagnetti A., D’Amato R., Persampieri T., Lawniczak M.K.N., Nolan T., Papathanos P.A. (2017). Rapid evolution of female-biased genes among four species of Anopheles malaria mosquitoes. Genome Res..

[24] Champagne D.E., Smartt C.T., Ribeiro J.M., James A.A. (1995). The salivary gland-specific apyrase of the mosquito Aedes aegypti is a member of the 5′-nucleotidase family. Proc. Natl. Acad. Sci. USA.

[25] Choumet V., Carmi-Leroy A., Laurent C., Lenormand P., Rousselle J.C., Namane A., Roth C., Brey P.T. (2007). The salivary glands and saliva of Anopheles gambiae as an essential step in the Plasmodium life cycle: A global proteomic study. Proteomics.

[26] Ribeiro J.M., Valenzuela J.G. (1999). Purification and cloning of the salivary peroxidase/catechol oxidase of the mosquito Anopheles albimanus. J. Exp. Biol..

[27] Gutierrez L.A., Gomez G.F., Gonzalez J.J., Castro M.I., Luckhart S., Conn J.E., Correa M.M. (2010). Microgeographic genetic variation of the malaria vector Anopheles darlingi root (Diptera: Culicidae) from Cordoba and Antioquia, Colombia. Am. J. Trop. Med. Hyg..

[28] Rodrigues P.T., Valdivia H.O., de Oliveira T.C., Alves J.M.P., Duarte A., Cerutti-Junior C., Buery J.C., Brito C.F.A., de Souza J.C., Hirano Z.M.B. (2018). Human migration and the spread of malaria parasites to the New World. Sci. Rep..

[29] Hemingway J., Shretta R., Wells T.N., Bell D., Djimde A.A., Achee N., Qi G. (2016). Tools and Strategies for Malaria Control and Elimination: What Do We Need to Achieve a Grand Convergence in Malaria?. PLoS Biol..

[30] Maldonado-Ruiz L.P., Montenegro-Cadena L., Blattner B., Menghwar S., Zurek L., Londono-Renteria B. (2019). Differential Tick Salivary Protein Profiles and Human Immune Responses to Lone Star Ticks (Amblyomma americanum) From the Wild vs. a Laboratory Colony. Front. Immunol..

[31] Ben Hadj Ahmed S., Chelbi I., Kaabi B., Cherni S., Derbali M., Zhioua E. (2010). Differences in the salivary effects of wild-caught versus colonized Phlebotomus papatasi (Diptera: Psychodidae) on the development of zoonotic cutaneous leishmaniasis in BALB/c mice. J. Med. Entomol..

[32] Fontaine A., Diouf I., Bakkali N., Misse D., Pages F., Fusai T., Rogier C., Almeras L. (2011). Implication of haematophagous arthropod salivary proteins in host-vector interactions. Parasit Vectors.

[33] Carlson J.C., Dyer L.A., Omlin F.X., Beier J.C. (2009). Diversity cascades and malaria vectors. J. Med. Entomol..

[34] Marie A., Ronca R., Poinsignon A., Lombardo F., Drame P.M., Cornelie S., Besnard P., Le Mire J., Fiorentino G., Fortes F. (2015). The Anopheles gambiae cE5 salivary protein: A sensitive biomarker to evaluate the efficacy of insecticide-treated nets in malaria vector control. Microbes Infect..

[35] Yoshiga T., Hernandez V.P., Fallon A.M., Law J.H. (1997). Mosquito transferrin, an acute-phase protein that is up-regulated upon infection. Proc. Natl. Acad. Sci. USA.

[36] Rawal R., Vijay S., Kadian K., Adak T., Pande V., Sharma A. (2018). Comparative proteomics of salivary glands of Anopheles culicifacies mosquitoes using tandem mass tag (TMT) mass spectrometry. J. Vector Borne Dis..

[37] Rodriguez M.H., Hernandez-Hernandez Fde L. (2004). Insect-malaria parasites interactions: The salivary gland. Insect Biochem. Mol. Biol..

[38] Wells M.B., Andrew D.J. (2019). Anopheles Salivary Gland Architecture Shapes Plasmodium Sporozoite Availability for Transmission. mBio.

[39] Das S., Radtke A., Choi Y.J., Mendes A.M., Valenzuela J.G., Dimopoulos G. (2010). Transcriptomic and functional analysis of the Anopheles gambiae salivary gland in relation to blood feeding. BMC Genom..

[40] Gutierrez L.A., Naranjo N.J., Cienfuegos A.V., Muskus C.E., Luckhart S., Conn J.E., Correa M.M. (2009). Population structure analyses and demographic history of the malaria vector Anopheles albimanus from the Caribbean and the Pacific regions of Colombia. Malar. J..

[41] DSSA Dirección Seccional de Salud de Antioquia. Eventos de Interés en Salud Pública Por Subregiones y Municipios. Antioquia 2007–2017. http://www.dssa.gov.co/index.php/estadisticas/eventos-en-salud-publica/item/71-eventos-de-interes-en-salud-publica-por-subregiones-y-municipios-antioquia-2007-2012.

[42] Singh B., Bobogare A., Cox-Singh J., Snounou G., Abdullah M.S., Rahman H.A. (1999). A genus—And species-specific nested polymerase chain reaction malaria detection assay for epidemiologic studies. Am. J. Trop. Med. Hyg..

[43] Burtnick M.N., Brett P.J., DeShazer D. (2014). Proteomic analysis of the Burkholderia pseudomallei type II secretome reveals hydrolytic enzymes, novel proteins, and the deubiquitinase TssM. Infect. Immun..

[44] Hurst K.E., Lawrence K.A., Essman M.T., Walton Z.J., Leddy L.R., Thaxton J.E. (2019). Endoplasmic Reticulum Stress Contributes to Mitochondrial Exhaustion of CD8(+) T Cells. Cancer Immunol. Res..

[45] Bronisz A., Wang Y., Nowicki M.O., Peruzzi P., Ansari K., Ogawa D., Balaj L., De Rienzo G., Mineo M., Nakano I. (2014). Extracellular vesicles modulate the glioblastoma microenvironment via a tumor suppression signaling network directed by miR-1. Cancer Res..

[46] Londono-Renteria B., Drame P.M., Weitzel T., Rosas R., Gripping C., Cardenas J.C., Alvares M., Wesson D.M., Poinsignon A., Remoue F. (2015). An. gambiae gSG6-P1 evaluation as a proxy for human-vector contact in the Americas: A pilot study. Parasit Vectors.

